# 6,6′-Di­nitro-1,1′-(ethane-1,2-di­yl)di(1*H*-indazole)

**DOI:** 10.1107/S1600536814004516

**Published:** 2014-03-05

**Authors:** Assoman Kouakou, El Mostapha Rakib, Abdelghani El Malki, Mohamed Saadi, Lahcen El Ammari

**Affiliations:** a Laboratoire de Chimie Organique et Analytique, Université Sultan Moulay Slimane, Faculté des Sciences et Techniques, Béni-Mellal, BP 523, Morocco; b Laboratoire de Chimie du Solide Appliquée, Faculté des Sciences, Université Mohammed V-Agdal, Avenue Ibn Battouta, BP 1014, Rabat, Morocco

## Abstract

The mol­ecule of the title compound, C_16_H_12_N_6_O_4_, is built up from two fused five- and six-membered rings linked by an ethyl­ene group. The dihedral angle between the planes through the indazole ring systems is 39.74 (5)°. The nitro groups are tilted by 7.2 (2) and 8.5 (2)° with respect to planes of the fused-ring systems. In the crystal, mol­ecules are linked by C—H⋯N and C—H⋯O hydrogen bonds into chains running parallel to the *c* axis.

## Related literature   

For biological activities of the indazole moiety, see: Ali *et al.* (2012 ▶); Abbassi *et al.* (2012 ▶); Plescia *et al.* (2010 ▶); Lee *et al.* (2001 ▶); Liu *et al.* (2011 ▶). For related compounds, see: Kouakou *et al.* (2013 ▶); Chicha *et al.* (2013 ▶).
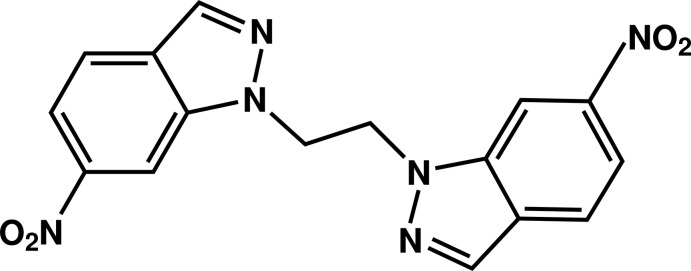


## Experimental   

### 

#### Crystal data  

C_16_H_12_N_6_O_4_
*M* *_r_* = 352.32
Monoclinic, 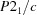

*a* = 9.410 (5) Å
*b* = 12.064 (5) Å
*c* = 14.804 (4) Å
β = 109.01 (2)°
*V* = 1588.9 (12) Å^3^
*Z* = 4
Mo *K*α radiation
μ = 0.11 mm^−1^
*T* = 296 K
0.37 × 0.32 × 0.26 mm

#### Data collection  

Bruker X8 APEX diffractometer
16446 measured reflections
3503 independent reflections
2667 reflections with *I* > 2σ(*I*)
*R*_int_ = 0.033

#### Refinement  

*R*[*F* ^2^ > 2σ(*F* ^2^)] = 0.039
*wR*(*F* ^2^) = 0.111
*S* = 1.03
3503 reflections
236 parameters
H-atom parameters constrained
Δρ_max_ = 0.20 e Å^−3^
Δρ_min_ = −0.19 e Å^−3^

### 

Data collection: *APEX2* (Bruker, 2009 ▶); cell refinement: *SAINT* (Bruker, 2009 ▶); data reduction: *SAINT*; program(s) used to solve structure: *SHELXS97* (Sheldrick, 2008 ▶); program(s) used to refine structure: *SHELXL97* (Sheldrick, 2008 ▶); molecular graphics: *ORTEP-3 for Windows* (Farrugia, 2012 ▶); software used to prepare material for publication: *PLATON* (Spek, 2009 ▶) and *publCIF* (Westrip, 2010 ▶).

## Supplementary Material

Crystal structure: contains datablock(s) I. DOI: 10.1107/S1600536814004516/rz5107sup1.cif

Structure factors: contains datablock(s) I. DOI: 10.1107/S1600536814004516/rz5107Isup2.hkl

Supporting information file. DOI: 10.1107/S1600536814004516/rz5107Isup3.cml

CCDC reference: 988920

Additional supporting information:  crystallographic information; 3D view; checkCIF report

## Figures and Tables

**Table 1:** Hydrogen-bond geometry (Å, °)

*D*—H⋯*A*	*D*—H	H⋯*A*	*D*⋯*A*	*D*—H⋯*A*
C7—H7⋯N5^i^	0.93	2.48	3.344 (2)	154
C15—H15⋯O1^i^	0.93	2.47	3.401 (2)	179

Symmetry code: (i) 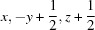.

**Fig. 1.**
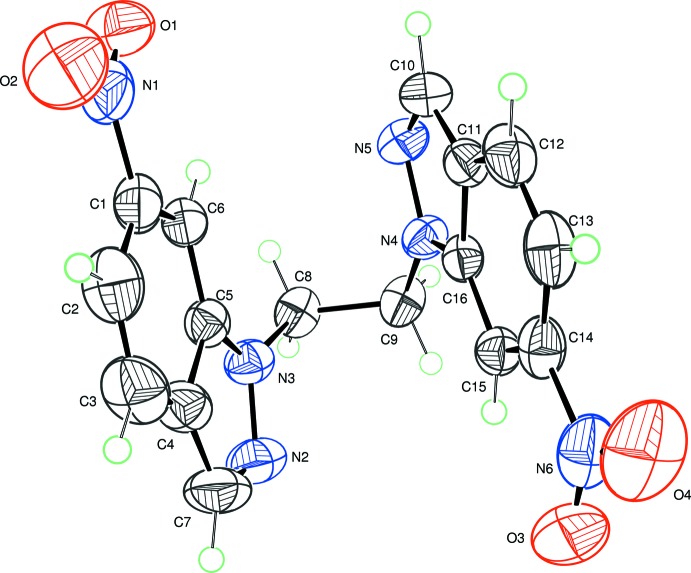
The molecular structure of the title compound with displacement ellipsoids drawn at the 50% probability level. H atoms are represented as small circles.

**Fig. 2.**
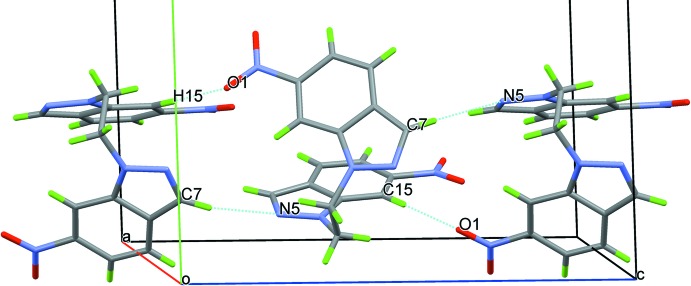
Partial crystal packing of the title compound showing a chain of molecules linked by hydrogen bonds (dashed lines).

## supplementary crystallographic information

### 1. Comment

Indazole moiety have been nucleus is a pharmaceutically important and emerging heterocycle with a broad spectrum of activities including anti-microbial1, anti-cancer, anti-inflammatory, anti- platelet, and selective 5-HT6 antagonists (Ali *et al.*, 2012; Abbassi *et al.*, 2012; Plescia *et al.*, 2010; Lee *et al.*, 2001; Liu *et al.*, 2011). The present work is a continuation of the investigation on the indazole derivatives published recently by our team (Kouakou *et al.*, 2013; Chicha *et al.*, 2013).

The molecule of the title compound is formed by two fused five- and six-membered rings linked by an ethylene group and connected to two nitro groups as shown in Fig. 1. The two fused ring systems (N2/N3/C1–C7 and N4/N5C10–C16) make dihedral angles of 7.2 (2)° and 8.5 (2)° with the planes through the attached nitro groups (N1/O1/O2 and N6/O3(O4), respectively. The dihedral angle between the indazole ring systems is 39.74 (5)°. In the crystal, molecules are linked by C7—H7···N5 and C15—H15···O1 hydrogen interactions (Table 1) into chains running parallel to the [0 0 1] direction as shown in Fig. 2.

### 2. Experimental

A solution of 6-nitroindazole (0.5 g, 3.06 mmol) and KOH (0.17 g, 3.08 mmol) in EtOH (15 ml) was heated under reflux for 48 h. The mixture was cooled and the solvent removed from the filtrate *in vacuo*. The formed 6-nitroindazole potassium salt and 1,2-ethylene dibromide (0.27 ml, 1.48 mmol) was heated in dimethylformamide (5 ml) under reflux for 3 h. The mixture was then cooled, all volatiles were removed *in vacuo* and water was added. The precipitate was filtered and was purified by column chromatography (EtOAc/hexane 2:8 *v*/*v*). The title compound was recrystallized from acetone (yield: 35%; m. p.: 468 K).

### 3. Refinement

H atoms were located in a difference Fourier map and treated as riding, with C–H = 0.93-0.97 Å and with *U*_iso_(H) = 1.2 *U*_eq_(C). One outlier (0 1 1) was omitted in the last cycles of refinement.

### Crystal data

**Table:**

C_16_H_12_N_6_O_4_	*F*(000) = 728
*M**_r_* = 352.32	*D*_x_ = 1.473 Mg m^−^^3^
Monoclinic, *P*2_1_/*c*	Mo *K*α radiation, λ = 0.71073 Å
Hall symbol: -P 2ybc	Cell parameters from 3672 reflections
*a* = 9.410 (5) Å	θ = 1.5–27.1°
*b* = 12.064 (5) Å	µ = 0.11 mm^−^^1^
*c* = 14.804 (4) Å	*T* = 296 K
β = 109.01 (2)°	Block, colourless
*V* = 1588.9 (12) Å^3^	0.37 × 0.32 × 0.26 mm
*Z* = 4	

### Data collection

**Table:**

Bruker X8 APEX diffractometer	2667 reflections with *I* > 2σ(*I*)
Radiation source: fine-focus sealed tube	*R*_int_ = 0.033
Graphite monochromator	θ_max_ = 27.1°, θ_min_ = 2.8°
φ and ω scans	*h* = −12→12
16446 measured reflections	*k* = −15→15
3503 independent reflections	*l* = −18→18

### Refinement

**Table:**

Refinement on *F*^2^	Secondary atom site location: difference Fourier map
Least-squares matrix: full	Hydrogen site location: inferred from neighbouring sites
*R*[*F*^2^ > 2σ(*F*^2^)] = 0.039	H-atom parameters constrained
*wR*(*F*^2^) = 0.111	*w* = 1/[σ^2^(*F*_o_^2^) + (0.0493*P*)^2^ + 0.3594*P*] where *P* = (*F*_o_^2^ + 2*F*_c_^2^)/3
*S* = 1.03	(Δ/σ)_max_ < 0.001
3503 reflections	Δρ_max_ = 0.20 e Å^−^^3^
236 parameters	Δρ_min_ = −0.19 e Å^−^^3^
0 restraints	Extinction correction: *SHELXL97* (Sheldrick, 2008), Fc^*^=kFc[1+0.001xFc^2^λ^3^/sin(2θ)]^-1/4^
Primary atom site location: structure-invariant direct methods	Extinction coefficient: 0.0033 (10)

### Special details

**Table:**

Geometry. All e.s.d.'s (except the e.s.d. in the dihedral angle between two l.s. planes) are estimated using the full covariance matrix. The cell e.s.d.'s are taken into account individually in the estimation of e.s.d.'s in distances, angles and torsion angles; correlations between e.s.d.'s in cell parameters are only used when they are defined by crystal symmetry. An approximate (isotropic) treatment of cell e.s.d.'s is used for estimating e.s.d.'s involving l.s. planes.
Refinement. Refinement of *F*^2^ against all reflections. The weighted *R*-factor *wR* and goodness of fit *S* are based on *F*^2^, conventional *R*-factors *R* are based on *F*, with *F* set to zero for negative *F*^2^. The threshold expression of *F*^2^ > σ(*F*^2^) is used only for calculating *R*-factors(gt) *etc*. and is not relevant to the choice of reflections for refinement. *R*-factors based on *F*^2^ are statistically about twice as large as those based on *F*, and *R*- factors based on all data will be even larger.

### Fractional atomic coordinates and isotropic or equivalent isotropic displacement parameters (Å^2^)

**Table:**

	*x*	*y*	*z*	*U*_iso_*/*U*_eq_	
O1	0.72668 (16)	0.44512 (14)	0.20575 (10)	0.0753 (4)	
O2	0.8642 (2)	0.57503 (14)	0.28985 (14)	0.1037 (6)	
O3	1.06798 (18)	0.14604 (14)	0.75890 (9)	0.0817 (5)	
O4	1.28535 (16)	0.17800 (15)	0.74730 (11)	0.0990 (6)	
N1	0.78171 (17)	0.49484 (13)	0.28105 (13)	0.0617 (4)	
N2	0.53003 (16)	0.25102 (14)	0.54188 (9)	0.0580 (4)	
N3	0.54014 (13)	0.24766 (11)	0.45233 (8)	0.0435 (3)	
N4	0.74940 (12)	0.09097 (10)	0.40649 (8)	0.0384 (3)	
N5	0.74567 (15)	0.09998 (12)	0.31430 (8)	0.0487 (3)	
N6	1.15060 (18)	0.16124 (12)	0.71175 (11)	0.0612 (4)	
C1	0.74301 (17)	0.45822 (13)	0.36515 (12)	0.0474 (4)	
C2	0.7928 (2)	0.52228 (15)	0.44873 (15)	0.0630 (5)	
H2	0.8535	0.5839	0.4515	0.076*	
C3	0.7517 (2)	0.49382 (17)	0.52569 (14)	0.0669 (5)	
H3	0.7817	0.5367	0.5809	0.080*	
C4	0.66362 (18)	0.39900 (15)	0.52037 (11)	0.0504 (4)	
C5	0.62089 (15)	0.33505 (12)	0.43641 (10)	0.0386 (3)	
C6	0.65760 (16)	0.36455 (12)	0.35590 (10)	0.0407 (3)	
H6	0.6263	0.3234	0.2997	0.049*	
C7	0.6008 (2)	0.34107 (18)	0.58152 (12)	0.0645 (5)	
H7	0.6090	0.3644	0.6429	0.077*	
C8	0.49431 (15)	0.14827 (13)	0.39587 (11)	0.0439 (4)	
H8A	0.4747	0.1659	0.3290	0.053*	
H8B	0.4017	0.1208	0.4030	0.053*	
C9	0.61375 (15)	0.05839 (13)	0.42568 (11)	0.0443 (4)	
H9A	0.6375	0.0436	0.4934	0.053*	
H9B	0.5748	−0.0094	0.3912	0.053*	
C10	0.87881 (19)	0.13663 (14)	0.31726 (11)	0.0505 (4)	
H10	0.9067	0.1501	0.2635	0.061*	
C11	0.97408 (16)	0.15298 (12)	0.41201 (10)	0.0401 (3)	
C12	1.12332 (17)	0.18656 (13)	0.45625 (13)	0.0511 (4)	
H12	1.1830	0.2074	0.4199	0.061*	
C13	1.17921 (16)	0.18815 (13)	0.55331 (13)	0.0520 (4)	
H13	1.2785	0.2087	0.5841	0.062*	
C14	1.08659 (16)	0.15870 (12)	0.60685 (11)	0.0433 (4)	
C15	0.93939 (15)	0.12611 (12)	0.56797 (10)	0.0375 (3)	
H15	0.8802	0.1076	0.6052	0.045*	
C16	0.88544 (14)	0.12277 (11)	0.46851 (9)	0.0337 (3)	

### Atomic displacement parameters (Å^2^)

**Table:**

	*U*^11^	*U*^22^	*U*^33^	*U*^12^	*U*^13^	*U*^23^
O1	0.0816 (9)	0.0892 (11)	0.0650 (9)	0.0071 (8)	0.0377 (7)	0.0152 (8)
O2	0.1124 (13)	0.0726 (11)	0.1501 (16)	−0.0232 (10)	0.0756 (12)	0.0175 (10)
O3	0.0884 (10)	0.1023 (12)	0.0437 (7)	0.0028 (9)	0.0066 (7)	−0.0094 (7)
O4	0.0647 (9)	0.1077 (13)	0.0874 (11)	−0.0184 (8)	−0.0262 (8)	−0.0118 (9)
N1	0.0585 (9)	0.0505 (9)	0.0863 (12)	0.0087 (7)	0.0376 (8)	0.0203 (8)
N2	0.0521 (8)	0.0823 (11)	0.0423 (8)	0.0015 (8)	0.0193 (6)	0.0077 (7)
N3	0.0397 (6)	0.0518 (8)	0.0396 (7)	−0.0015 (6)	0.0139 (5)	0.0034 (5)
N4	0.0345 (6)	0.0445 (7)	0.0350 (6)	−0.0009 (5)	0.0096 (5)	0.0047 (5)
N5	0.0539 (8)	0.0556 (8)	0.0348 (7)	0.0022 (6)	0.0122 (5)	0.0045 (6)
N6	0.0602 (9)	0.0482 (9)	0.0565 (9)	−0.0003 (7)	−0.0065 (7)	−0.0092 (7)
C1	0.0448 (8)	0.0398 (9)	0.0594 (10)	0.0049 (7)	0.0195 (7)	0.0067 (7)
C2	0.0607 (11)	0.0428 (10)	0.0824 (13)	−0.0098 (8)	0.0189 (9)	−0.0067 (9)
C3	0.0704 (12)	0.0604 (12)	0.0614 (12)	−0.0067 (9)	0.0098 (9)	−0.0233 (9)
C4	0.0484 (8)	0.0589 (11)	0.0403 (8)	0.0030 (8)	0.0096 (7)	−0.0072 (7)
C5	0.0340 (7)	0.0411 (8)	0.0386 (7)	0.0026 (6)	0.0088 (6)	0.0006 (6)
C6	0.0425 (8)	0.0388 (8)	0.0402 (8)	0.0044 (6)	0.0126 (6)	0.0003 (6)
C7	0.0645 (11)	0.0929 (15)	0.0362 (9)	0.0050 (10)	0.0164 (8)	−0.0053 (9)
C8	0.0307 (7)	0.0476 (9)	0.0490 (8)	−0.0051 (6)	0.0068 (6)	0.0043 (7)
C9	0.0341 (7)	0.0446 (9)	0.0510 (9)	−0.0058 (6)	0.0095 (6)	0.0098 (7)
C10	0.0589 (10)	0.0556 (10)	0.0445 (9)	0.0084 (8)	0.0272 (7)	0.0085 (7)
C11	0.0410 (7)	0.0369 (8)	0.0481 (8)	0.0058 (6)	0.0221 (6)	0.0066 (6)
C12	0.0387 (8)	0.0456 (9)	0.0772 (12)	0.0017 (7)	0.0300 (8)	0.0100 (8)
C13	0.0307 (7)	0.0395 (9)	0.0805 (12)	−0.0024 (6)	0.0110 (7)	0.0025 (8)
C14	0.0396 (7)	0.0328 (8)	0.0494 (9)	0.0009 (6)	0.0035 (6)	−0.0035 (6)
C15	0.0376 (7)	0.0354 (7)	0.0399 (7)	0.0015 (6)	0.0132 (6)	0.0017 (6)
C16	0.0304 (6)	0.0311 (7)	0.0397 (7)	0.0020 (5)	0.0117 (5)	0.0027 (5)

### Geometric parameters (Å, º)

**Table:**

O1—N1	1.223 (2)	C4—C7	1.416 (3)
O2—N1	1.220 (2)	C5—C6	1.391 (2)
O3—N6	1.216 (2)	C6—H6	0.9300
O4—N6	1.221 (2)	C7—H7	0.9300
N1—C1	1.474 (2)	C8—C9	1.520 (2)
N2—C7	1.309 (3)	C8—H8A	0.9700
N2—N3	1.3603 (18)	C8—H8B	0.9700
N3—C5	1.3643 (19)	C9—H9A	0.9700
N3—C8	1.445 (2)	C9—H9B	0.9700
N4—N5	1.3581 (17)	C10—C11	1.411 (2)
N4—C16	1.3650 (18)	C10—H10	0.9300
N4—C9	1.4494 (19)	C11—C12	1.402 (2)
N5—C10	1.316 (2)	C11—C16	1.4082 (19)
N6—C14	1.472 (2)	C12—C13	1.360 (2)
C1—C6	1.367 (2)	C12—H12	0.9300
C1—C2	1.404 (2)	C13—C14	1.401 (2)
C2—C3	1.361 (3)	C13—H13	0.9300
C2—H2	0.9300	C14—C15	1.373 (2)
C3—C4	1.400 (3)	C15—C16	1.3929 (19)
C3—H3	0.9300	C15—H15	0.9300
C4—C5	1.406 (2)		
			
O2—N1—O1	123.51 (18)	C4—C7—H7	123.8
O2—N1—C1	118.09 (18)	N3—C8—C9	111.77 (12)
O1—N1—C1	118.37 (15)	N3—C8—H8A	109.3
C7—N2—N3	105.94 (14)	C9—C8—H8A	109.3
N2—N3—C5	111.41 (13)	N3—C8—H8B	109.3
N2—N3—C8	119.09 (13)	C9—C8—H8B	109.3
C5—N3—C8	128.28 (12)	H8A—C8—H8B	107.9
N5—N4—C16	111.41 (11)	N4—C9—C8	111.39 (12)
N5—N4—C9	118.85 (11)	N4—C9—H9A	109.3
C16—N4—C9	129.58 (12)	C8—C9—H9A	109.3
C10—N5—N4	106.31 (12)	N4—C9—H9B	109.3
O3—N6—O4	123.09 (18)	C8—C9—H9B	109.3
O3—N6—C14	118.95 (15)	H9A—C9—H9B	108.0
O4—N6—C14	117.96 (18)	N5—C10—C11	111.71 (13)
C6—C1—C2	124.04 (16)	N5—C10—H10	124.1
C6—C1—N1	117.51 (15)	C11—C10—H10	124.1
C2—C1—N1	118.44 (16)	C12—C11—C16	119.62 (14)
C3—C2—C1	119.66 (17)	C12—C11—C10	136.07 (14)
C3—C2—H2	120.2	C16—C11—C10	104.29 (13)
C1—C2—H2	120.2	C13—C12—C11	118.78 (14)
C2—C3—C4	118.92 (16)	C13—C12—H12	120.6
C2—C3—H3	120.5	C11—C12—H12	120.6
C4—C3—H3	120.5	C12—C13—C14	119.75 (14)
C3—C4—C5	119.45 (16)	C12—C13—H13	120.1
C3—C4—C7	136.90 (17)	C14—C13—H13	120.1
C5—C4—C7	103.63 (16)	C15—C14—C13	124.32 (15)
N3—C5—C6	130.81 (13)	C15—C14—N6	117.30 (15)
N3—C5—C4	106.64 (13)	C13—C14—N6	118.38 (14)
C6—C5—C4	122.54 (15)	C14—C15—C16	114.97 (13)
C1—C6—C5	115.31 (14)	C14—C15—H15	122.5
C1—C6—H6	122.3	C16—C15—H15	122.5
C5—C6—H6	122.3	N4—C16—C15	131.16 (12)
N2—C7—C4	112.36 (15)	N4—C16—C11	106.28 (12)
N2—C7—H7	123.8	C15—C16—C11	122.54 (13)
			
C7—N2—N3—C5	1.38 (17)	C5—N3—C8—C9	87.72 (18)
C7—N2—N3—C8	169.77 (14)	N5—N4—C9—C8	−68.45 (17)
C16—N4—N5—C10	0.40 (17)	C16—N4—C9—C8	106.61 (16)
C9—N4—N5—C10	176.31 (14)	N3—C8—C9—N4	−65.19 (17)
O2—N1—C1—C6	−176.34 (16)	N4—N5—C10—C11	−0.10 (18)
O1—N1—C1—C6	5.4 (2)	N5—C10—C11—C12	177.90 (17)
O2—N1—C1—C2	5.0 (2)	N5—C10—C11—C16	−0.21 (18)
O1—N1—C1—C2	−173.22 (15)	C16—C11—C12—C13	0.6 (2)
C6—C1—C2—C3	−2.2 (3)	C10—C11—C12—C13	−177.33 (17)
N1—C1—C2—C3	176.39 (16)	C11—C12—C13—C14	−1.3 (2)
C1—C2—C3—C4	1.7 (3)	C12—C13—C14—C15	0.8 (2)
C2—C3—C4—C5	0.6 (3)	C12—C13—C14—N6	179.97 (14)
C2—C3—C4—C7	178.7 (2)	O3—N6—C14—C15	−7.5 (2)
N2—N3—C5—C6	−179.39 (14)	O4—N6—C14—C15	172.00 (16)
C8—N3—C5—C6	13.6 (2)	O3—N6—C14—C13	173.18 (16)
N2—N3—C5—C4	−0.72 (16)	O4—N6—C14—C13	−7.3 (2)
C8—N3—C5—C4	−167.77 (13)	C13—C14—C15—C16	0.6 (2)
C3—C4—C5—N3	178.52 (15)	N6—C14—C15—C16	−178.62 (12)
C7—C4—C5—N3	−0.18 (17)	N5—N4—C16—C15	−179.32 (14)
C3—C4—C5—C6	−2.7 (2)	C9—N4—C16—C15	5.3 (3)
C7—C4—C5—C6	178.62 (14)	N5—N4—C16—C11	−0.53 (15)
C2—C1—C6—C5	0.2 (2)	C9—N4—C16—C11	−175.88 (14)
N1—C1—C6—C5	−178.41 (12)	C14—C15—C16—N4	177.23 (14)
N3—C5—C6—C1	−179.27 (14)	C14—C15—C16—C11	−1.4 (2)
C4—C5—C6—C1	2.2 (2)	C12—C11—C16—N4	−178.06 (13)
N3—N2—C7—C4	−1.5 (2)	C10—C11—C16—N4	0.43 (15)
C3—C4—C7—N2	−177.3 (2)	C12—C11—C16—C15	0.9 (2)
C5—C4—C7—N2	1.1 (2)	C10—C11—C16—C15	179.35 (13)
N2—N3—C8—C9	−78.47 (16)		

### Hydrogen-bond geometry (Å, º)

**Table:**

*D*—H···*A*	*D*—H	H···*A*	*D*···*A*	*D*—H···*A*
C7—H7···N5^i^	0.93	2.48	3.344 (2)	154
C15—H15···O1^i^	0.93	2.47	3.401 (2)	179

Symmetry code: (i) *x*, −*y*+1/2, *z*+1/2.
